# Education for values and bioethics

**DOI:** 10.1186/s40064-015-0815-z

**Published:** 2015-02-01

**Authors:** Rui Nunes, Ivone Duarte, Cristina Santos, Guilhermina Rego

**Affiliations:** Faculty of Medicine of the University of Porto (Portugal-EU), Estrada da Circunvalação, n. 9925, 4250-150 Porto, EU Portugal

**Keywords:** Active learning, Bioethics, Teaching, Values

## Abstract

“Education for Values and Bioethics” is a project which aims to help the student to build his/her personal ethics. It was addressed to ninth grade students (mean age 14) who frequented public education in all schools of the City of Porto, Portugal-EU in 2010–2013 (N-1164). This research and action project intended to promote the acquisition of knowledge in the following areas: interpersonal relationships, human rights, responsible sexuality, health, environment and sustainable development, preservation of public property, culture, financial education, social innovation and ethical education for work. The students were asked to answer to a knowledge questionnaire on bioethics. To assess the values it was used Leonard Gordon’s Survey of Personal Values and Survey of Interpersonal Values. The results of this study show that the project contributes to an increase of knowledge in the area of bioethics. Also the students enrolled in the program showed a development with regards the acquisition of the basic values of pluralistic societies. It is also suggested that this general knowledge on bioethics could be especially helpful to students that want a career in health sciences.

## Introduction

In a pluralistic society increasingly marked by constant technological and cultural developments, only citizens capable of critical thinking may respond to the challenges that constantly arise (Engelhardt 1996). It is through the active participation of the youths in their community and the promotion of a reflection about oneself, of others and the world around them, that autonomous, participative and civically responsible citizenship can be promoted.

In this context, as the school is a special area of learning social skills, it is essential to implement a comprehensive education that promotes a personal, social, emotional and cultural development of each child (Curtler 2004). Thus, education for bioethics may be an important contribution to this goal, in a transversal perspective and in accordance with a wide scope of topics ranging from human rights to health education, or environmental ethics which constitute the emerging concerns of today’s society. Once the human being is the central reference in society, education should become a phenomenon of interpersonal relationships whose content are values, information, knowledge, feelings, attitudes and skills, which aim to promote the development of a full human person.

In this regard, a specific project concerning education for bioethics was implemented, addressed to 9th grade students (mean age 14) who frequented public education in all schools of the City of Porto, Portugal, in the academic years 2010/2011, 2011/2012 e 2012/2013. This project was born of a partnership between the Department of Bioethics of the Faculty of Medicine of the University of Porto, of the Portuguese Association of Bioethics and of the Porto City Council, having been enrolled all schools of the city. “Education for Values and Bioethics” is a specific training project which aims to help each youth build his/her personal ethics, not only in the perspective of the group to which he/she belongs, but in a wider form showing the surrounding world. Alerting the student to important ethical dilemmas such as end-of-life decisions, reprogenetics, environmental protection, gender stereotypes and discrimination, and to establish a link between the emergence of rights and correlative duties to those rights may contribute to the promotion of conscious, responsible and participatory citizens. Sensitize and educate the youths were also objectives of this project aiming their active participation in improving their ethical, social, emotional, cognitive and behavioural skills.

The specific objective of this paper is to determine if it is possible to promote in 9th grade students a universal culture of human rights through the diffusion of knowledge, skills and attitude change. And, also, promote self-knowledge and self-determination in interpersonal relationships. Another objective was to assess whether there are significant differences between the students subjected and not subjected to training regarding the knowledge as well as the change of values that shape their personality.

## Theory and method

The development of teaching and learning in the field of bioethics has followed the general development of modern societies. For a long time now the teaching of healthcare professions follows a body of universal ethical principles, and a core curriculum of bioethics was developed for global application. Teaching of bioethics is present today in undergraduate level, postgraduate and ongoing training of physicians and other healthcare professionals (Myser 1998). However, the values and principles underlying bioethics and biomedical ethics can and should be taught in the formative phase of the youngsters, namely in elementary school (Levinson and Reiss 2003; Macer 2006).

Bioethics aims the creation of a new unitary knowledge through interdisciplinarity. To achieve this goal bioethics necessarily have to resort to generalizations (as the concept of quality of life), universal values (such as the individual right to self-determination), and also to its own paradigms, such as the principles of Beauchamp and Childress (Beauchamp and Childress 2012). But, although quality of life or even the right to self-determination are self-explaining concepts other issues, such as Beauchamp and Childress principlism, are more related to a way of achieving consensus in a global society were cultural pluralism prevails. The elaboration of a set of guiding principles, of systematic application, in clinical practice and in human research, which would be midway between the fundamental ethical theory – integrated body of rules and principles – and rules of conduct is a practical way to analyse ethical dilemmas both in teaching and in clinical practice.

To specify the goals of teaching and learning bioethics is perhaps the most important task in organizing a program for elementary school students. From there, naturally, the program content will take place, as well as the methods and materials necessary for attaining these goals. The aim is to help the student learn how to achieve those goals. However, specifying objectives will evoke, eventually, another issue, which is whether it is possible to teach and learn bioethics. Thus, learning objectives may include, from a cognitive point of view, the attempt to:

1. Increase the sensitivity of the student to ethical issues in biomedicine, environment and biodiversity;
2. Promote critical reflection on values of personal and professional nature and of society in general;
3. Identify the ethical principles underlying decision making;
4. Learning the structure of ethical analysis of situations as well as its justification in the conceptual plan;
5. Allow a critical and systematic ethical decision approach in the specific context.

Beyond the goals of cognitive nature it is also important the acquisition of behavioural objectives, i.e. a specific interaction in the field of bioethics. For example, it is important that the student knows how to overcome the gap between theory and practice, and has sufficient flexibility to accept the free expression of others, with tolerance and respect. If, on one hand, bioethics is founded on ethical principles more or less shared by pluralistic societies (Engelhardt 1996), and materialized in international conventions (National Commission for the Protection of Human Subjects of Biomedical and Behavioural Research 1978; UNESCO 2005), on the other, students hope, as in other areas of education, that teachers strongly defend a specific thesis, presenting the arguments which they consider necessary and appropriate. Indeed, it is expected that the teacher is not just a neutral presenter (presenting the various perspectives about a certain topic), but that he/she proposes a logical, coherent and reasoned argument.

Another essential objective of bioethics teaching is to teach the student to be an active partner in learning, using several logical reasoning models which stimulate critical reflection on the fundamental ethical issues. But, it is also important to consider that ethical theory, like any theory, can be reformulated according to the development of society (Rachels 1999).

As mentioned before, the affective dimension of interpersonal relationships should not be neglected. This dimension is the least explored aspect in bioethics teaching, because, being subjective, it is difficult to measure, although it is possible to evaluate the standing of a student by discussing case-problems. Training assumes then a fundamental role, as it enables the transmission and acquisition of knowledge.

To evaluate the acquisition of knowledge and skills is an essential task in teaching and learning bioethics (Savulescu et al. 1999). There is some consensus on the strategy to adopt in relation to an objective measurement of knowledge acquisition in the cognitive and behavioural sphere (Consensus Statement 1998). It is at stake the knowledge of the principles and underlying concepts as well as the ability to recognize and deal with ethical problems. Among several possible methods of evaluation the following were suggested (Doyal 1993): written reports on specific topics or case-problems; written tests, in the form of short questions, multiple choice or true and false; questionnaires; self-directed exercises; role-playing; group discussion; live discussion; and oral exam answering to moral dilemmas posed by examiners. These different strategies show that, at least, cognitive data may be taught and learned. The development of skills at the level of behaviour, in terms of its relational dimension, can also be taught, although with more difficulty (Mitchell et al. 1994).

However, the fact that it is possible to teach and assess the behavioural dimension, does not mean that either the knowledge or behaviours will be reflected in future behaviour. Choosing a program faces the same type of difficulties found in the formulation of the pedagogical processes and in assessing its results. All these methods require deep thought because, in such a sensitive area as bioethics one must set up a curriculum of education that expresses universally accepted principles but that escapes the ancestral temptation to indoctrinate, to shape the human mind in accordance with certain ethical assumptions. That is, once again, to reach a consensus on core ethical issues and how to transmit them. Hence, ten thematic areas were selected which were considered as core to a bioethics program in the 9th grade.

The role of the teaching staff is to present the different opinions allowing a wide scope of interpretation by taking into account the different cultural perspectives. Donna Dickenson points out that the role of a bioethics teacher should be (Dickenson and Parker 2000): clearly define the problem; articulate the different points of view; argue for a point of view making use of relevant experiences in this field; make an effort to understand the opposing positions; possibly revise earlier positions; and promote consensus. It should be recognized, however, that despite the teaching of bioethics is widespread throughout all developed countries there are important programmatic differences between different cultures and even within countries (Claudot et al. 2007).

From a methodologically perspective this project is an action research study considered as an interactive process, focusing on one issue, which invests in promoting and monitoring routes of experimentation, on existing practices in each context, aiming its improvement and where all participants are actors and authors of the project. Assessment practices provide important information about the nature and conditions of training, about the established goals and eventual contradictions, while contributing to its reconstruction. This project, of close articulation between evaluation and education involved bilaterally trainers and trainees. As a methodological asset of this study it should be emphasized the group of teachers, comprising young university teachers with training on bioethics that, despite the age difference, can still be generally considered as a peer group. The peer group exerts a strong social influence on young people, playing a key role in the construction and consolidation of the identity and autonomy process, sharing ideas, attitudes, values and behaviours.

This project had the intention to educate for values and bioethics using hybrid educational resources that promote personal and social development of the individual contributing to a healthy, individualized, informed and responsible living, as well as the acquisition of knowledge in the following specific areas:

1. Interpersonal relationships;
2. Education for human rights;
3. Education for responsible sexuality;
4. Education for health;
5. Education for environment and sustainable development;
6. Education for the preservation and defence of public property;
7. Education for culture;
8. Financial education;
9. Innovation and social entrepreneurship;
10. Education for work.

The project was implemented along three consecutive years with an average participation of 18 elementary schools of the City of Porto, Portugal, EU. It began in October 2010 and its completion took place in June 2013. Its implementation began by performing one or more 90 minutes weekly training actions during school hours, on each of the ten thematic units. The methodology developed includes training modules, to be distributed in 16 sessions of 90 minutes each, coordinated by specialists in each area of education (a total training of 16 × 90 minutes per student) having as a goal the raising awareness among young people about the promotion of humanistic values and full citizenship. The project integrated 9th grade students from different social strata and also included 9th grade students with severe to profound deafness, from a school of reference in this area. In this particular class, teachers had the presence of a sign language interpreter in all sessions.

It is accepted today that instead of providing large amounts of information it should be highlighted the positive and negative aspects of a particular subject, behaviour, or feature (Rivers et al. 2008), as teenagers do not have enough life experience, basing their decisions on simplified representations of information. Thus, the ten thematic units from the project “Education for Values and Bioethics” fill known gaps in the school curriculum and maybe another contribution to the formation of more informed, involved and responsible citizens.

Peer education is a methodology that allows, at the same time, to promote the learning and development of oneself and others through the development of rational, intentional, systematic, fundamental and technical actions. The peers are considered as an invaluable human resource to the extent that they enable better adjustment of the messages. The teachers were specifically prepared not only to address the thematic content but also the ability to assertively communicate and to work the group as a team in its innovative spirit and openness to change.

To evaluate the results of this action research project the students were asked to answer, individually to a knowledge questionnaire and two surveys on values, at two different moments. The knowledge questionnaire was constructed based on the syllabus of each of the ten modules that constitutes the project. The purpose of this instrument is to measure the level of knowledge that both groups have on the issues addressed both at the beginning of the project and at the end of it. But this action research has also as an aim to evaluate acquisition/change in values as they are fundamental in determining what people do and how they do it. Many of the immediate decisions of the individual as well as their long-term plans are influenced, consciously or unconsciously, by their system of values.

One way to assess the values of individuals is to determine the relative importance that individuals attribute to the various activities. In this sense two surveys were used, the Survey of Personal Values (SPV) and the Survey of Interpersonal Values (SIV) (Gordon 1960; 1967). The Personal Values and Interpersonal Values Surveys are both validated for the Portuguese language (Gordon 2001a, b). They are applied in adolescents and adults in school, clinical, or research contexts. Both are easy to apply, with an approximate duration of fifteen minutes each. The Survey of Personal Values aims to evaluate certain critical values that helps determine how individuals deal with their day-to-day problems.

Indeed, to measure values that “determine the manner in which an individual copes with the problems of everyday living” Gordon developed the Survey of Personal Values (Gordon 1967). This survey projects six scores:

1. Practical Mindedness: To always get one’s money’s worth, to take good care of one’s property, to get full use out of one’s possessions, to do things that will pay off, to be very careful with one’s money.
2. Achievement: To work on difficult problems, to have a challenging job to tackle, to strive to accomplish something significant, to set the highest standards of accomplishment for oneself, to do an outstanding job in anything one tries.
3. Variety: To do things that are new and different, to have a variety of experiences, to be able to travel a great deal, to go to strange or unusual places, to experience an element of danger.
4. Decisiveness: To have strong and firm convictions, to make decisions quickly, to always come directly to the point, to make one’s position on matters very clear, to come to a decision and stick to it.
5. Orderliness: To have well-organized work habits, to keep things in their proper place, to be a very orderly person, to follow a systematic approach in doing things according to a schedule.
6. Goal Orientation: To have a definite goal toward which to work, to stick to a problem until it is solved, to direct one’s attention toward clear-cut objectives, to know precisely where one is headed, to keep one’s goals clearly in mind.

The Survey of Interpersonal Values (Gordon 1960) is a self-report designed to measure values involving the individual’s relationships to other people or their relationships to him/her across six scales:

1. Support: Being treated with understanding, receiving encouragement from other people, being treated with kindness and consideration.
2. Conformity: Doing what is socially correct, following regulations closely, doing what is accepted and proper, being a conformist.
3. Recognition: Being looked up to and admired, being considered important, attracting favourable notice, achieving recognition.
4. Independence: Having the right to do whatever one wants to do, being free to make one’s own decisions, being able to do things in one’s own way.
5. Benevolence: Doing things for other people, sharing with others, helping the unfortunate, being generous.
6. Leadership: Being in charge of other people, having authority over others, being in a position of leadership or power.

The filling in of the questionnaires was done individually and voluntarily, and confidentiality was strictly guaranteed. According to Portuguese policy the study did require review by an ethics committee. The project was submitted and approved by the Ethics Committee of the Faculty of Medicine of the University of Porto, in compliance with the ethical norms and guidelines for research involving human beings. All the procedures were performed in accordance with the last revision of the Helsinki Declaration of the World Medical Association.

## Results

This action research comprised the evaluation of two distinct groups of students (the students enrolled and the controls), and of the teachers and principals from the schools included in the project. Three different evaluations were performed:

- At the end of each year they were asked to answer a questionnaire on satisfaction and evaluation of the project (N-1164). The relative frequencies of students and teachers who rated the training as, sufficient, good or very good was calculated.
- A knowledge evaluation in two different moments. The comparison of the mean knowledge between the first and second moment was performed with paired sample *t*-tests in both control and training groups.
- A values evaluation in two different moments. The results of the surveys at first were described with means and respective standard deviations. The differences between the second and first moment were calculated in the various dimensions of the inventories, these differences were compared between the control and training groups using the independent sample *t*-test. A statistical significance level of 0.05 was used.

The results show that globally this study was considered a very positive initiative for the students enrolled in this project, being clear the high degree of satisfaction, corresponding to global expectations (the sum of “Good” with “Very Good” cover more than 90% of the responses). In turn, the majority of the teachers that belonging to the school staff did not participate in this action research (but still followed the project) considered that the project meets their expectations, that the issues addressed were properly integrated and that it constitutes an important initiative in the education of young people. Mentioning also that their perception about the students’ involvement was positive and very good, and 70% of these teachers reported the existence of positive feedback from students.

With regards knowledge acquisition, although there has been, in all years, a significant increase in knowledge both in training groups as in control groups, the increase was always more pronounced in the training classes with the exception of the control class of the school year of 2010/2011. In the training classes there is always an average increase of 10% (2 out of 20) or more, while in the control groups, when rises significantly, the climb is around 5% (1 out of 20) (Table 1).

**Table 1:** **Comparison of the mean “knowledge” in the first and second time in both control and training groups**

		**First time**		**Second time**		**p**
	**n**	**mean**	**Sd**	**mean**	**Sd**	
**2010/2011**						
Training group	241	11.6	2.3	13.6	2.3	*<0.001*
Control group	199	11.2	2.3	11.5	2.5	0.224
**2011/2012**						
Training group	312	10.9	2.6	13.7	2.5	*<0.001*
Control group	248	10.6	2.7	11.7	2.4	*<0.001*
**2012/2013**						
Training group	361	10.5	2.8	13.5	2.6	*<0.001*
Control group	326	10.4	2.3	11.3	2.3	*<0.001*

sd : standard deviation.

The values evaluation comprised a sample of 1393 answers to the SPV, 722 of whom were female (52%), with a mean age of 14.3 years and a standard deviation of 0.8 years in the first moment. In the year 2010/2011 the sample has 353 answers, in the year 2011/2012 it has 442 answers, and in the year 2012/2013 it has 598 answers. Of the 1366 responses to SIV, 771 were in training classes (55%). The SIV and SPV means and standard deviations in the first moment, by gender, are described in Table 2.

**Table 2:** **SIV and SPV means and standard deviations (sd) in the first moment, by gender**

	**Female**		**Male**	
	**Mean**	**SD**	**Mean**	**SD**
SIV				
Support	16.12	4.24	15.59	4.33
Conformity	17.03	5.63	16.93	5.78
Recognition	9.17	4.08	10.34	4.56
Independence	18.64	5.39	17.87	5.82
Benevolence	19.81	4.81	17.75	5.32
Leadership	8.72	4.94	10.96	5.50
SPV				
Practical mindedness	13.25	3.87	14.77	3.92
Achievement	15.21	3.57	15.49	3.85
Variety	10.51	6.64	9.45	6.61
Decisiveness	13.75	4.24	12.83	4.18
Orderliness	16.67	4.47	16.71	4.76
Goal orientation	20.06	4.55	20.12	4.66

sd – standard deviation.

There are no statistically significant differences between the control and the training class in the mean difference between the second and first moment, for scales that constitute the SIV (Table 3). But a significant difference was found between the control class and the training class in the difference between the second and the first moment in the SPV “practical mindedness”; the training class has risen from the first to the second moment while the control class had even a small decrease (p = 0.015).

**Table 3:** **Mean differences between the second and first moment in the various dimensions of inventories in both control and training groups**

	**Training group**		**Control group**		
	**Mean**	**SD**	**Mean**	**SD**	**p**
SIV					
Support	−0.12	4.50	0.13	4.71	0.304
Conformity	−0.58	5.29	−1.05	5.33	0.108
Recognition	0.17	4.81	0.15	4.67	0.946
Independence	0.82	5.46	0.78	5.13	0.894
Benevolence	−0.65	5.23	−0.50	5.28	0.604
Leadership	0.28	5.68	0.55	5.37	0.376
SPV					
Practical mindedness	0.56	4.52	−0.02	4.38	*0.015*
Achievement	−0.31	4.18	0.20	4.15	*0.023*
Variety	0.85	6.30	0.62	6.42	0.514
Decisiveness	0.20	4.45	0.76	4.60	*0.023*
Orderliness	−0.51	4.60	−0.97	4.53	0.060
Goal orientation	−0.75	5.03	−0.45	5.37	0.274

sd – standard deviation.

There was also a significant difference between the control class and the training class in the difference between the second and the first moment in the SPV “achievement”, having the training class decreased from the first to the second moment while the control group had an increase (p = 0.023). A significant difference was found between the control class and the training class in the difference between the second moment and the first in the SPV “decisiveness”. Both classes have risen, but the control group increased more than the training class from the first to the second moment (p = 0.023).

## Discussion

A controversial issue is whether it is possible to improve the students’ character or if, on the other hand, the teaching of bioethics should only aim to provide intellectual resources for the student to develop and improve his/her ethical behaviour (Stern 2000). To encourage an active partnership between teachers and students it should be acknowledged the existence of two types of student concerning learning styles. This differentiation occurs at different levels depending on the extent by which it is assessed (Tobias 1990): type of information perception, type of information perceived, way of organizing information, way of processing information, way followed for understanding.

According to Felder this dichotomy is neither absolute nor static and may evolve and change over the course of time (Felder 1993). However, it is important that teachers and students take into account the existence of several different ways of learning that will be decisive in the quantity and quality of knowledge acquired. And through a joint and shared partnership in the classroom the objective might be to stimulate the student to become a citizen and a professional who can acknowledge the ethical issues and the underlying values. A proper education enables teaching and learning to be self-motivated, and directed both to the resolution of specific problems and to acquire differentiated skills. Moreover, it stimulates the interaction, responsibility and collaboration with colleagues. By forming teams one learns better because, in an effort to teach a colleague, attention and unfold interdependence is promoted. These groups, preferably heterogeneous and randomly formed, can be maintained throughout all the teaching period (Pattison et al. 1999; Nilstun et al. 2001; Mattick and Bligh 2006).

The results of this study allow to conclude that the project contributes to an increase of knowledge in the area of bioethics by students who completed training when compared with students in the control group. The rise in the control classes after the second year of the project may be due, among other factors, to the fact that teachers (those belonging to the school staff and not participating in bioethics teaching but who closely followed the project in their school) can reproduce in the other classes where they teach (controls) some of the subjects discussed using the adopted methodology (besides their natural ripening process).

With regard to the results of the Survey of Personal Values with statistical significance they seem to indicate that young people enrolled in the project go on to develop a more reflective and mature thinking in comparison to an absolutist or immediate thinking that is usual at this age. What until then seemed to be black or white, easy to decide, starts to have other shades. On the other hand students of control groups’ value more rapid decisions probably because they hold firm and strong convictions and the mechanisms that fuel their attitudes appear to be more influenced by the momentum than reasoning. Throughout the training, it has been developed a critical and reflective spirit which is somehow opposed to the “decisiveness” dimension, i.e. possess strong and firm beliefs, making decisions quickly. Moreover, as the teenager matures, he/she becomes less impulsive and less drawn to immediate rewards (Steinberg 2009).

Regarding the “practical mindedness”, i.e., “take good care of their own; take advantage of what you have; do something that compensates, give the best use to your money”, training class students have a higher tendency for their appreciation, which is in accordance with what has been thought in the thematic units of the project. The results show that for students in training classes the dimension of “achievement” is not so valued, namely the importance of success, as they start to consider other dimensions of their personal value system.

Concerning the Survey of Interpersonal Values, the authors did not find statistically significant differences between the training classes and the control classes, which may be due to the fact that adolescents in this age group are more focused on their personal values over interpersonal ones, which may be explained by the moral stage of development in which they are. Adolescence is characterized as a stage for construction of social values and teenagers aspiring moral perfection tend to progressively express great altruism. And increased knowledge allowing the development of new cognitive skills will allow him/her to mentally prepare hypotheses, discuss ideas and confront opinions, building his/her own theory of reality.

Robert Selman contributed to the explanation of how children develop their skills by positioning oneself in the perspective of the “other” who is involved in a given social situation. With cognitive development, the person adopts complex viewpoints of differentiation: between oneself and others, between oneself and different people in a group and between oneself and groups or social systems (Selman 1980).

Analysing the results it can be concluded that the project was positive for the students, noting their high satisfaction level and the very positive impact on the knowledge and development of a critical way of thinking. Students have by the end of the action research a greater knowledge of basic ethical values such as individual rights and responsibilities of all human beings. Young people develop their contacts with others, in school, in leisure contexts and family, and, within the latter, there is less and less time available for the relationship and communication between parents and children. Promoting opportunities for the young to face ethical dilemmas at school, and thus developing cognitive, emotional and behavioural skills, is an important step if personal and interpersonal values are to be developed (Raths et al. 1966).

## Conclusion

In the 20th century, bioethics was a prerogative of both scholars interested in this topic and health professionals who daily faced ethical dilemmas raised by life sciences (Reich 1999). However, at the beginning of the 21st century it is considered that most of the dilemmas of bioethics are indeed ethical concerns of all citizens, so it is only natural to expose to elementary school students the values and principles of a global bioethics so that they can build their personality in an ethically structured way (Brousseau and Mirk 1997; Center for Bioethics High school bioethics project 2002). Different experiences which exist in this field should be compared at an international scale so that consensual guidelines on curriculum and method of teaching/learning on elementary education in bioethics can be suggested.

According to the National Institutes of Health there are four important reasons to teach bioethics in the high school: advance students’ science understanding, prepare students to make informed, thoughtful choices, promote respectful dialogue among people with diverse views and to cultivate critical-reasoning skills (National Institutes of Health 2009). It follows that this general knowledge on bioethics could be especially helpful to students that want a career in health sciences because the high school student begins very early to think as a bioethicist does and this is a critical factor for traditional healthcare professions such as medicine or nursing, or even to a geneticists or a bioengineer (Kennedy Institute of Ethics 2002).

Encouraging adolescents to discuss different topics, such as abortion, environmental protection, or gender equality, may contribute to the development of useful skills, essential to their success as persons and autonomous citizens. This is a responsibility shared by the school, the family and the community.
